# Demand for Prophylaxis after Bioterrorism-Related Anthrax Cases, 2001

**DOI:** 10.3201/eid1101.040272

**Published:** 2005-01

**Authors:** Edward A. Belongia, Burney Kieke, Ruth Lynfield, Jeffrey P. Davis, Richard E. Besser

**Affiliations:** * Marshfield Clinic Research Foundation, Marshfield, Wisconsin, USA; † Minnesota Department of Health, Minneapolis, Minnesota, USA; ‡ Wisconsin Division of Public Health, Madison, Wisconsin, USA; § Centers for Disease Control and Prevention, Atlanta, Georgia, USA

**Keywords:** research, anthrax, fluoroquinolone, prophylaxis, drug resistance, bioterrorism

## Abstract

Media reports suggested increased public demand for anthrax prophylaxis after the intentional anthrax cases in 2001, but the magnitude of anthrax-related prescribing in unaffected regions was not assessed. We surveyed a random sample of 400 primary care clinicians in Minnesota and Wisconsin to assess requests for and provision of anthrax-related antimicrobial agents. The survey was returned by 239 (60%) of clinicians, including 210 in outpatient practice. Fifty-eight (28%) of those in outpatient practice received requests for anthrax-related antimicrobial agents, and 9 (4%) dispensed them. Outpatient fluoroquinolone use in both states was also analyzed with regression models to compare predicted and actual use in October and November 2001. Fluoroquinolone use as a proportion of total antimicrobial use was not elevated, and anthrax concerns accounted for an estimated 0.3% of all fluoroquinolone prescriptions. Most physicians in Minnesota and Wisconsin managed anthrax-related requests without dispensing antimicrobial agents.

## Abstract 2

In 1991, most physicians in Minnesota and Wisconsin managed patients concerns about anthrax without dispensing prophylactic antimicrobial agents.

Until recently, human anthrax infections in the United States have been rare and generally limited to agricultural workers with exposure to infected animals or animal products. The first bioterrorism-related cases of human anthrax occurred in late 2001, when spore-laden envelopes were mailed to news media facilities and government officials. Twenty-two cases of anthrax were identified from October 4 to November 20, including 5 fatalities (*1*). Cases occurred in New York, New Jersey, Pennsylvania, Virginia, Maryland, Connecticut, and Florida. The patients included targeted persons, people who worked with targeted persons, postal workers, and people who were exposed along the mail route of spore-containing envelopes.

At the affected sites, ≈10,000 people were advised to take antimicrobial prophylaxis for at least 60 days to prevent anthrax, and an estimated 32,000 people initiated antibiotic prophylaxis (*1*,*2*). Most persons received initial prophylaxis with a fluoroquinolone (ciprofloxacin), a class of drug that is also important for treatment of community-acquired pneumonia and other serious infections (*3*,*4*). In public announcements regarding anthrax, the Centers for Disease Control and Prevention (CDC) emphasized the overall low risk to the population but also endorsed actions to minimize personal risk. These measures included not opening suspicious mail, keeping mail away from the face when opened, and washing hands after handling mail (*5*). Although national publicity generated a high level of public concern throughout the country, no evidence of anthrax spore release was found in any other regions of the United States.

Media reports in late 2001 reflected a high level of anxiety in the general public regarding anthrax. An ABC News/Washington Post poll in mid-October 2001 found that 65% of respondents were worried about letters contaminated with the anthrax bacteria, and 54% were worried about an anthrax attack on themselves, their close friends, or relatives (*6*). At the same time, anecdotal evidence suggested that ciprofloxacin sales were increasing at some pharmacies (*7*,*8*). New Web sites marketed ciprofloxacin “prevention packs” to anxious consumers (*9*). However, the magnitude of public demand for anthrax-related antimicrobial agents and for physicians to dispense them has not been assessed in regions of the country where no cases or exposures occurred.

Inappropriate use of fluoroquinolones raises concern for several reasons. They can potentially cause adverse reactions, and excessive hoarding could contribute to drug shortages. Fluoroquinolones are critical drugs for managing a variety of serious infections, and in recent years the rate of resistance has increased among both gram-positive and gram-negative organisms because of the increasing use of these agents (*10*–*12*). Recent fluoroquinolone use is an independent risk factor for fluoroquinolone-resistant pneumococcal infections (*13*).

The purpose of this investigation was to assess patient demand for anthrax prophylaxis and changes in fluoroquinolone use in late 2001 in a region of the country in which no cases or exposures occurred. Clinicians were surveyed regarding patient demand for anthrax prophylaxis and use of prophylactic antimicrobial agents, and a commercial database was analyzed to assess changes in overall fluoroquinolone use.

## Methods

### Clinician Survey

A 1-page survey and cover letter were mailed to 400 primary care clinicians in Minnesota and Wisconsin in October 2002. Within each state, a simple random sample of 200 clinicians was selected from among those with an active medical license in any of the following specialties: emergency medicine, family practice, general practice, general internal medicine, pediatrics, nurse practitioner, or physician assistant. The sampling frame was obtained from the licensing agencies in each state and included 5,800 clinicians in Minnesota and 6,510 clinicians in Wisconsin. Two reminder letters were sent to all survey recipients to encourage participation.

The anonymous survey contained 12 questions to obtain information on specialty, county of practice, outpatient practice activity during the last quarter of 2001, requests for anthrax prophylaxis from October through December of 2001, and distribution of antimicrobials for anthrax prophylaxis or stockpiling. Clinicians who indicated that they prescribed or distributed anthrax-related antimicrobial agents were asked to specify the number and types of people (i.e., patients, family members, acquaintances, self) who received the drugs, and the specific drugs that were used. The occurrence of suspected anthrax-related exposures was not assessed. Clinicians who indicated they were not in full- or part-time outpatient medical practice from October through December of 2001 were asked to return the survey without answering the other questions.

The survey procedures were reviewed and approved by the institutional review board at Marshfield Clinic.

### Outpatient Use of Antimicrobial Agents

The primary measures of fluoroquinolone use were based on prescribing data (Xponent, IMS Health, Inc., Plymouth Meeting, PA) and volume retail distribution data (DDD, IMS Health, Inc.) for the states of Wisconsin and Minnesota. The prescribing and distribution data represent independent measures of antimicrobial use that were obtained to evaluate a statewide program to promote appropriate antimicrobial use. Prescribing data were available for the years 2000 through 2002. Volume distribution data were available for the years 1999 through 2002. For this analysis, oral antimicrobial agents were grouped into the following three categories: fluoroquinolones (ciprofloxacin, levofloxacin, lomefloxacin, moxifloxacin, ofloxacin), tetracyclines (doxycycline, tetracycline, minocycline), and other oral antimicrobial agents (amoxicillin, amoxicillin-clavulanate, ampicillin, cephalosporins, extended-spectrum macrolides, macrolides, penicillin, and trimethoprim-sulfamethoxazole). The first two categories represented drug classes that were most likely to be used for anthrax prophylaxis or stockpiling, since they were recommended as first-line agents by CDC (*14*). The third category provided a measure of seasonal and annual trends in outpatient use of antimicrobials, since the drugs in this category (with the possible exception of penicillin and amoxicillin) would not be appropriate for managing anthrax exposure. Penicillin or amoxicillin use was recommended for anthrax use only when the first-line agents were contraindicated, and it is therefore unlikely that a substantial number of these prescriptions were generated for anthrax-related concerns. Prescribing and volume distribution data were not available for individual drugs within each class. Only oral agents in solid (capsule or tablet) formulation were included in the analyses to avoid including prescriptions for young children.

Xponent prescribing data included all dispensed prescriptions in Minnesota or Wisconsin from licensed prescribers. The total number of prescriptions written in each state was available by month for the years 2000, 2001, and 2002. The prescribing databases were compiled by IMS Health with proprietary methods. The Xponent prescribing database was derived from transactional data provided by 59% of all retail pharmacies in Wisconsin and Minnesota, including 65% of chain pharmacies and 51% of independent retail pharmacies. Prescriptions from unsampled stores were estimated on the basis of prescription totals from matched nearby stores with weighting to adjust for differences in total retail sales volume, which was available for nearly all stores. Estimates were also weighted to account for the distance between sampled stores and matched unsampled stores, with closer stores contributing more to the estimated prescription volume. The proportion of all prescriptions in each state based on estimated data from unsampled stores was 33%–37%.

Retail distribution was measured based on the volume of antimicrobial agents distributed to retail outlets on a monthly basis for the years 1999 through 2002 (DDD data, IMS Health, Inc.). Retail distribution data (measured in kilograms) were ultimately derived from wholesalers and distributors serving pharmacies in both states. Volume was based on distribution to retailers rather than actual sales to patients, and distributed antimicrobial agents could be returned to the wholesalers without being sold. In this situation, returned antimicrobial agents were subtracted from the total distributed in a given month to yield the net retail distribution for each drug class. As a result, the retail distribution data may overestimate or underestimate actual distribution to patients, particularly in short time periods. Inpatient pharmacies, prisons, veterinarians, nursing homes, dialysis clinics, and federal government sites were excluded from the volume distribution data. The DDD database captured 93% of actual volume of antimicrobial distribution in these states.

### Statistical Analysis

To account for the state-level sampling in the clinician survey, analysis weights were generated for each survey responder. These weights were computed by multiplying the inverse of the sampling probability for each responder by the inverse of the response rate in the appropriate state. Weighted analyses for complex sample designs were performed by using the SAS 8.2 (SAS Institute Inc., Cary, NC) and SUDAAN 8.0 (RTI International, Research Triangle Park, NC) software. The impact of the weighting was minimal in our study; estimated percents for the weighted and unweighted analyses differed by <l percentage point. We therefore present unweighted point estimates, except for the estimate of anthrax-related antimicrobial drug prescribing. All statistical tests for the clinician survey were based on weighted data to reflect the complex sample design. Categorical variables were compared by using the chi-square test.

For the Xponent prescribing data, we fit models for the absolute number and proportion of prescriptions for fluoroquinolones and tetracyclines. Absolute numbers of prescriptions were modeled by using linear regression, while proportions of prescriptions were modeled by using negative binomial regression. All of the models contained categoric effects for year and month and indicator variables for October, November, and December of 2001. We generated predictions for the fourth quarter of 2001 by computing linear combinations of the appropriate parameters from the above models. The modeling procedures for the DDD data were the same as those for the Xponent data, except that we modeled total retail distribution volume by month for each drug category.

## Results

### Clinician Survey

Surveys were returned by 239 (60%) of 400 clinicians, including 123 in Wisconsin and 116 in Minnesota. Twenty-nine (12%) of the 239 respondents were excluded from subsequent analyses because they were not engaged in full-or part-time outpatient practice during the last 3 months of 2001. The respondent medical practices were located in 68 counties; 58 (28%) practices were located in the 11-county Minneapolis–St. Paul metropolitan area, and 24 (11%) were located in the 4-county Milwaukee-Waukesha metropolitan area. One hundred fifty-seven (75%) were physicians (MD or DO), and 52 (25%) were nurse practitioners or physician assistants. Physician specialties included family practice (42%), internal medicine (30%), pediatrics (18%), emergency medicine (4%), and other (6%).

Fifty-eight (28%) of the clinicians reported that someone had asked them to prescribe an antimicrobial drug to prevent anthrax or stockpile in case of future bioterrorist attacks. Physicians were significantly more likely than nonphysicians to receive requests for antimicrobial agents (Table). The occurrence of patient requests by state, practice specialty, or practice location (Minneapolis–St. Paul and Milwaukee–Waukesha metropolitan areas vs. other areas) did not differ significantly.

**Table:** Characteristics of Minnesota and Wisconsin clinicians who received requests for antimicrobial agents to prevent anthrax during the last quarter of 2001

	Received requests (%)	p value
Prescriber type		
Physician	52/157 (33)	<0.001
Nonphysician	6/52 (12)	
Practice location		
Metropolitan area	22/82 (27)	0.82
Nonmetropolitan area	36/128 (28)	
State		
Minnesota	29/105 (28)	1.0
Wisconsin	29/105 (28)	
Physician specialty*		
Family practice	26/62 (41)	0.61
Internal medicine	14/42 (30)	
Pediatrics	8/28 (28)	
Emergency medicine	2/5 (33)	

*Nine physicians who listed 2 specialties and 2 who listed “other” as a specialty were excluded from the frequency distribution for physician specialty.

Nine (4%) of the clinicians provided antimicrobial agents to 11 persons for anthrax prevention or stockpiling. Seven clinicians provided such agents for a single person, and 2 clinicians provided them for 2 persons. All 9 clinicians were MDs or DO, and 8 (89%) practiced in Wisconsin. Among 58 clinicians who received such requests, 8 (14%) provided them; 1 additional clinician did not receive requests but provided these drugs for family members. Nine (82%) of the 11 courses of antimicrobial agents were given to patients seen in the clinician’s practice, and 2 were given to family members. One clinician reported that prophylactic antimicrobial agents were given to a patient who had exposure to a building in Washington, D.C., where anthrax release was confirmed; the risk for anthrax exposure was unknown for the other 10 persons. A fluoroquinolone (levofloxacin or ciprofloxacin) was used for 10 (91%) of the 11 courses; amoxicillin was given to 1 person. No other classes of antimicrobial agents were used.

Survey responses were used to estimate the total number of anthrax-related fluoroquinolone prescriptions written in Wisconsin and Minnesota during the last quarter of 2001. State medical licensing records indicate that ≈10,807 primary care clinicians were engaged in full- or part-time outpatient practice in Minnesota and Wisconsin at that time. If the survey responses are representative of this group, an estimated 420 clinicians (3.9%) in these states provided anthrax-related antimicrobial agents. The 95% confidence limits (CI) for this proportion are 1.2% and 6.5%, corresponding to 135 and 706 clinicians, respectively. The total number of anthrax-related courses of antimicrobial agents prescribed during the last quarter of 2001 was estimated to be 523 (95% CI, 394–653) in the two states. If these prescriptions were mostly for fluoroquinolones, they would have represented ≈0.3% of all fluoroquinolone prescriptions written in Minnesota or Wisconsin from October through December of 2001.

### Outpatient Use of Antimicrobial Agents

**Figure 1:**
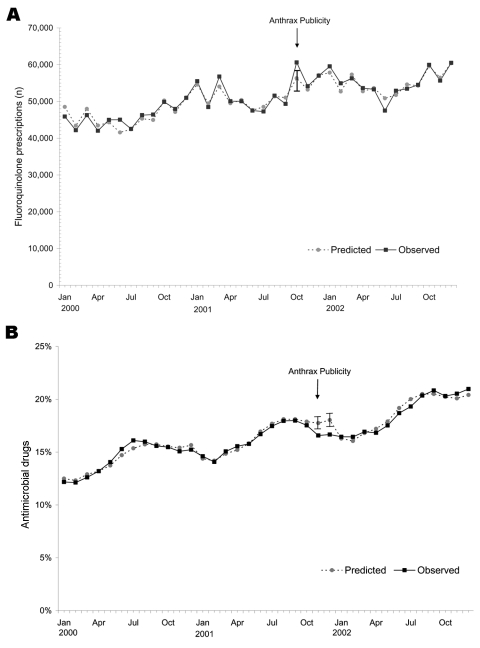
A) Actual and predicted fluoroquinolone prescriptions, January 2000 through December 2002. B) Actual and predicted fluoroquinolone prescriptions as a percentage of all outpatient antibiotic prescriptions, January 2000 through December 2002. Tetracycline and related antimicrobial agents were excluded from the denominator in each month. Vertical bars show 95% confidence intervals. All models included categorical effects for year and month and indicator variables for October, November, and December, 2001

**Figure 2:**
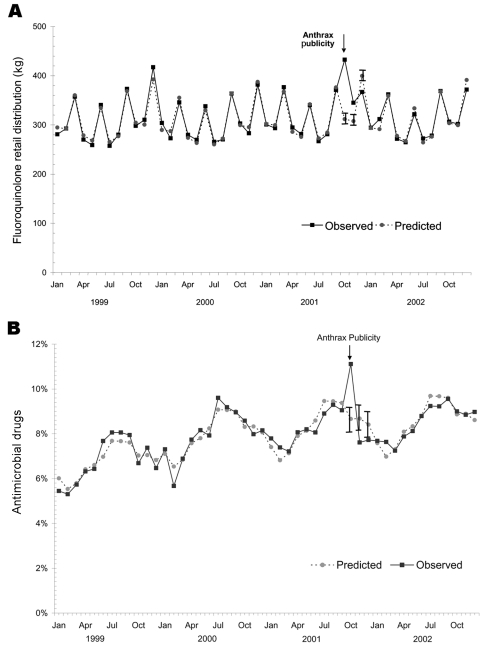
A) Retail distribution of fluoroquinolone antimicrobial agents, January 1999 through December 2002. Volume was measured in kilograms. B) Actual and predicted retail distribution of fluoroquinolone antimicrobial agents as a percentage of total antibiotic volume distribution (excluding tetracyclines), January 1999 through December 2002. Vertical bars show 95% confidence intervals.

The annual number of fluoroquinolone prescriptions in Minnesota and Wisconsin increased 20% from 2000 to 2002, while the annual number of prescriptions declined during this period for tetracycline drugs and other antimicrobials. In October 2001, the number of fluoroquinolone prescriptions was 22% higher than in October 2000, and the number of fluoroquinolone prescriptions exceeded the 95% CI based on the predictive model (Figure 1). The proportion of all antimicrobial prescriptions (excluding tetracyclines) represented by fluoroquinolones gradually increased from 2000 to 2002, and this proportion was higher in October 2001 (17.5%) than in October 2000 (15.5%). However, the observed proportion of fluoroquinolone prescriptions was lower than predicted in October and November of 2001, after adjusting for the secular trend (Figure 1). Retail distribution of oral fluoroquinolones was significantly elevated in October and November of 2001 (Figure 2). The fluoroquinolone proportion of total retail distribution of antimicrobial agents (excluding tetracyclines) also increased significantly in October 2001, but it was lower than predicted in November (Figure 2).

**Figure 3:**
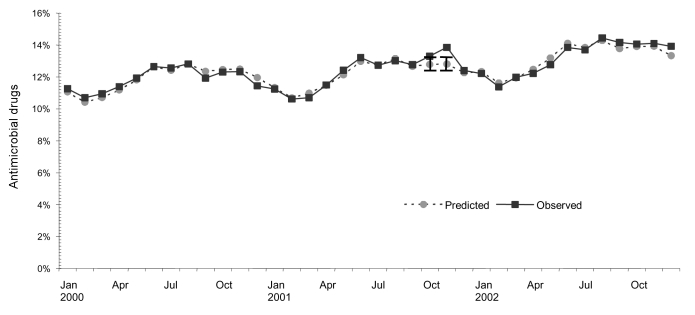
Actual and predicted tetracycline group prescriptions as a percentage of all outpatient antimicrobial prescriptions (excluding fluoroquinolones), January 2000 through December 2002. Vertical bars show 95% confidence intervals.

The proportion of all antimicrobial prescriptions (excluding fluoroquinolones) that were for tetracyclines was near the upper limit of the 95% CI in October 2001, and it exceeded the upper limit in November 2001 (Figure 3). The proportion of antimicrobial drug retail volume represented by tetracyclines was not elevated during those months (data not shown).

## Discussion

The results of this study confirm that public demand for anthrax-related antimicrobial agents was substantial in Minnesota and Wisconsin, since one fourth of primary care clinicians received requests for these drugs. We found that physicians received these requests more often than nonphysicians. Despite requests from patients and family members, relatively few antimicrobial agents were dispensed for anthrax prophylaxis. Extrapolation of survey responses to all primary care clinicians in Minnesota and Wisconsin suggests that ≈500 anthrax-related antimicrobial drug courses were dispensed during the last quarter of 2001. Even if nearly all of these were fluoroquinolones, the volume of anthrax-related fluoroquinolone use represented only a small fraction of total use in Minnesota and Wisconsin during that period.

The prescribing and retail distribution data showed surges in fluoroquinolone use during October 2001, but total use of antimicrobials also increased for unknown reasons during that period. Fluoroquinolone prescriptions as a proportion of all antimicrobial prescriptions were not elevated, which was consistent with the findings from the clinician survey. Although tetracycline/doxycycline prescriptions as a proportion of all outpatient antimicrobial prescriptions (excluding fluoroquinolones) were elevated in October and November 2001, the clinician survey indicated that this increase was unrelated to anthrax prescribing. None of the survey respondents reported using tetracycline/doxycycline for this purpose, and ciprofloxacin was the anthrax drug that received most media attention in late 2001.

Two other studies have addressed national use of antimicrobial agents following the anthrax cases in 2001. In one study that used a national pharmacy claims database, the rate of ciprofloxacin use increased 9.8% in October 2001 relative to October 2000 (*15*). As expected, the greatest increase in use was observed in New York, the mid-Atlantic states, and Florida. Ciprofloxacin prescribing rates were not reported for Minnesota or Wisconsin. In this study, the denominator was defined as the number of covered persons who filled a prescription for any drug or eligible health product during that month. As a result, the observed rate differences may have been influenced by both changes in the numerator (number of ciprofloxacin prescriptions) and changes in the denominator (number of persons filling any prescription).

A similar study used IMS Health National Prescription Audit Plus7 data to compare national ciprofloxacin use from July to December of 2001 and 2000 (*16*). Comparison drugs included oral azithromycin and cefuroxime, which are commonly used in outpatient practice but not recommended for anthrax prophylaxis. Ciprofloxacin prescriptions increased by 42%; cefuroxime prescriptions declined by 3%. The results were not reported by region, and they included prescriptions in New York, Florida, and other affected regions. The authors did not assess monthly ciprofloxacin prescriptions as a percentage of all antimicrobial prescriptions. The results of our study suggest that short-term variations in single drug prescribing should be interpreted with caution when the specific diagnoses or prescribing indications are not known. We found that a short-term increase in fluoroquinolone use in Minnesota and Wisconsin was accompanied by an overall increase in antimicrobial drug use. Thus, factors unrelated to anthrax may have also contributed to the observed increase in fluoroquinolone use during October 2001, especially in unaffected regions of the United States.

Whether patterns of antimicrobial use in Minnesota and Wisconsin are generalizable to other unaffected regions of the United States is not known. For example, total ciprofloxacin prescriptions in October 2001 appeared to increase >25% in some unaffected states, including Nevada, California, and New Mexico (*15*). No information is available regarding the clinical indications for these prescriptions, and how much of this increase can be attributed to anthrax-related prescribing is unclear. Other factors may also contribute to regional differences in prescribing, since physicians in the northeastern and southern United States are more likely to prescribe broad-spectrum antimicrobials than those in the midwestern or western regions (*17*).

The survey results in Minnesota and Wisconsin may have underestimated actual anthrax-related prescribing, since clinicians who dispensed antimicrobial agents may have been reluctant to return the survey. However, the cover letter and survey questions were neutral regarding the appropriateness of antimicrobial drug use, and the survey was anonymous. Poor recall is another potential source of error, since the survey was conducted approximately 1 year after the first cases of intentional anthrax occurred. Because we were asking about unusual events that were outside the scope of normal clinical practice, we assumed that clinicians would still recall any anthrax-related prescribing. Finally, the survey results did not allow us to determine if patients consumed these agents for anthrax prophylaxis, or if they were stockpiled for future use.

The human anthrax cases in 2001 and the related events illustrate how quickly demand for a critical drug can escalate as a result of heightened public anxiety and media attention. Most physicians in Minnesota and Wisconsin managed public and patient expectations without dispensing antimicrobial agents. However, social factors clearly influence prescribing decisions (*18*), and effective public and physician communication will be essential to promote rational behavior if similar or more extreme situations arise in the future. A communications strategy should be developed in advance that includes identifying key experts at the state and national level for news media communications and devising a plan for coordination and consistency of messages from different agencies.
